# Supplementary material for: Mineralocorticoid Receptor May Regulate Glucose Homeostasis through the Induction of Interleukin-6 and Glucagon-Like peptide-1 in Pancreatic Islets
Source: J Clin Med. 2019 May 14;8(5):674. doi: 10.3390/jcm8050674 (PMC6571682; doi:10.3390/jcm8050674)
Supplement: Supplementary file 1 [file jcm-08-00674-s001.pdf]

Table S1: List of qPCR primers

Gene	species	Forward primers	Reverse primers
GAPDH	mouse	TCACCACCATGGAGAAGGC	CCTAAGCAGTTGGTGGTGCA
GAPDH	human	CATGTTCCAATATGATTCCACC	CTCCATGGTGGTGAAGACGC
Caspase-3	mouse	TCTGACTGGAAAGCCGAAACTC	TTCCACTGTCTGTCTCAATAC
Bcl-2	mouse	GGCTACGAGTGGGATACTGGAG	AGGCTGGAAGGAGAAGATGC
MR	mouse	GCGTTCTTCTCTTGGACCTG	GCAAAATCCCAGACCGACTA
MR	human	GAAAGACGGTGGGGTCAAG	TGCTGAAGGCAAGGGAGTAG
GR	mouse	GGGCGCCAAGTGATTGCCGCAGT	CCAACCCAGGGCAAATGCCATGA
GR	human	GGGCGGCAAGTGATTGCAGCAGT	CCACCCCAGAGCAAATGCCATAA
IL-6	mouse	AGTTGCCTTCTTGGGACTGA	CTGTGAAGTCTCCTCTCCGG
IL-6	human	CCTAGAGTACCTCCAGAACAG	CTTCGTCAGCAGGCTGGCA
PC1/3	mouse	CATCTTTGTCTGGGCTTCAGG	GCTTGTTATTCGCTGGTCTGTG
Pdx-1	mouse	CTGCCACCATGAACAGTGAG	GATCCCAGCGAGCTTGTAAA
SGK1	mouse	TTGGGGCTGTCCTGTATGAG	GGTGCCTTGCCGAGTTTG

Table S2: List of siRNA sequences.

	sequence
siMR	CCCGCUCAACAUGCCGUCUUCAGUA
siGR	GCCAUUUCUGUUCAUGGCGUGAGUA
CONMR	CCCAGUACACGUUGCUUCACGCGUA
CONGR	GCCUCUUCAUCGGUAGUGCAUAGUA

siMR: siRNA targeting MR; siGR: siRNA targeting GR; CONMR: negative control siRNA (MR sense sequence); CONGR: negative control siRNA (GR sense sequence).

Table S3: List of qPCR primers and sense sequence for mutated MRE.

Gene	Forward primer	Reverse primer
IL-6 promoter	GTGGTACCCCCTAGTTGTGTCTTGC	CTGGAGGGGAGATAGAGCTTC

	hybridized oligonucleotides sequence
mutated MRE	GTGGTACCCCCTAGTTGTGTCTTGCCATTTTAAATTTTTTTTCATTTTTTAATTTTAATAAGGTTT CCAATCAGCCCCACCCGCTCTGGCCCCACCCTCACCCTCCAACAAAGATTTATCAAATGTGGG ATTTTCCCATGAGTCTCAATATTAGAGTCTCAACCCCCAATAAATATAGGACTGGAGATGTCTG AGGCTCATTCTGCCCTCGAGCCCACCGGGAACGAAAGAGAAGCTCTATCTCCCCTCCAG

Figure S1

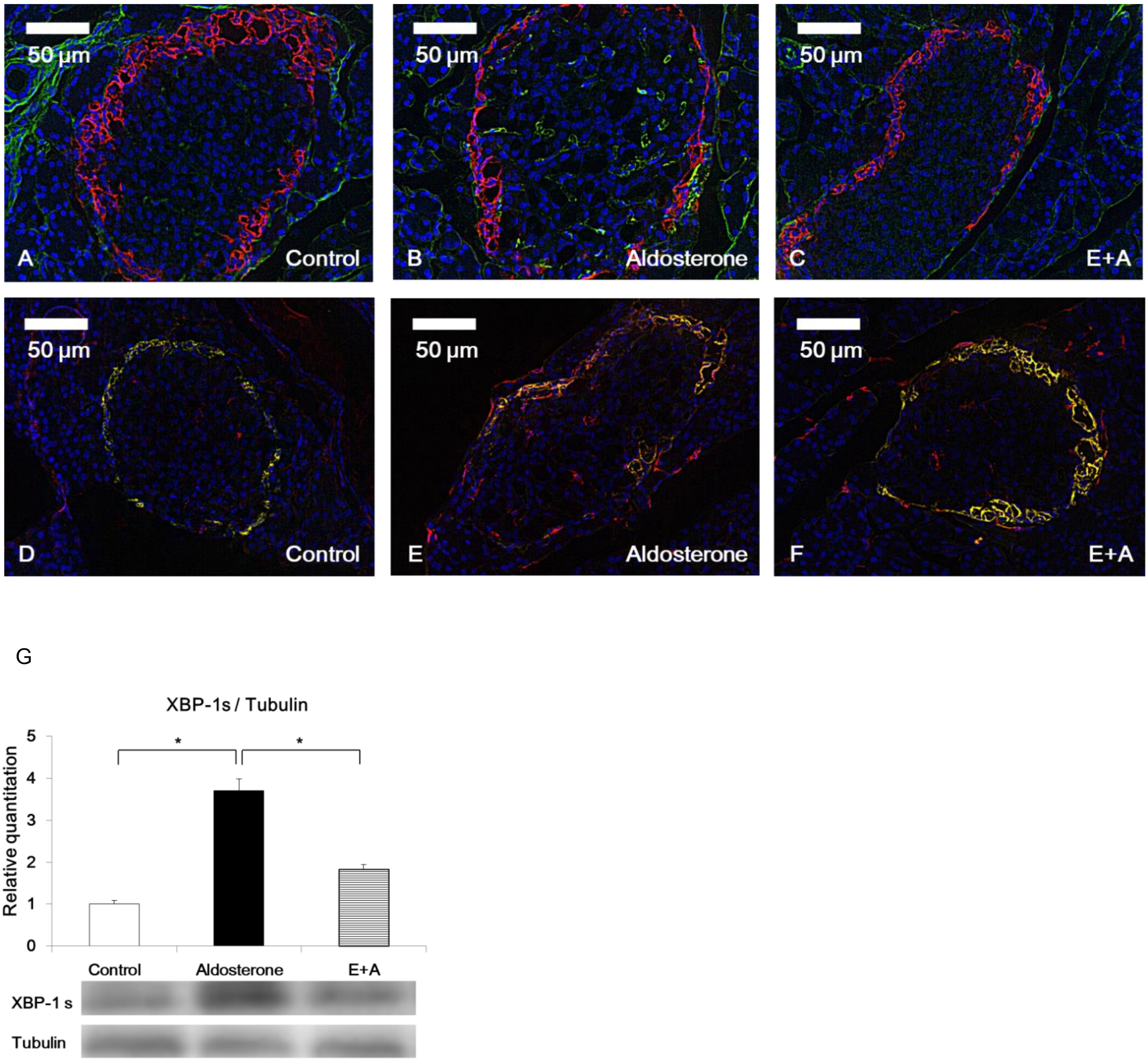
50 µm
A
Control
50 µm
B
Aldosterone
50 µm
C
E+A
50 µm
D
Control
50 µm
E
Aldosterone
50 µm
F
E+A
G
XBP-1s / Tubulin
Relative quantitation
0
1
2
3
4
5
*
*
Control
Aldosterone
E+A
XBP-1 s
Tubulin

Figure S2

A

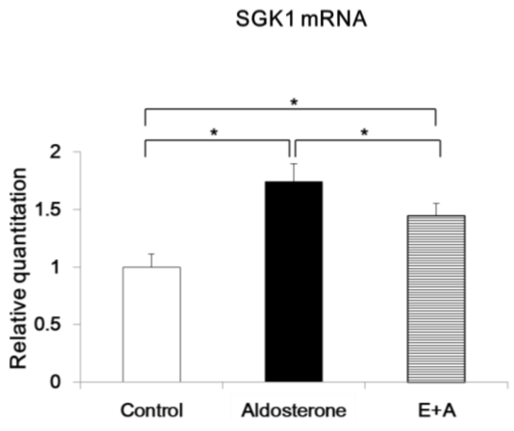

B

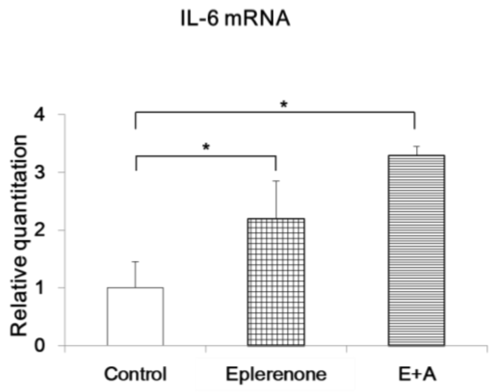

C

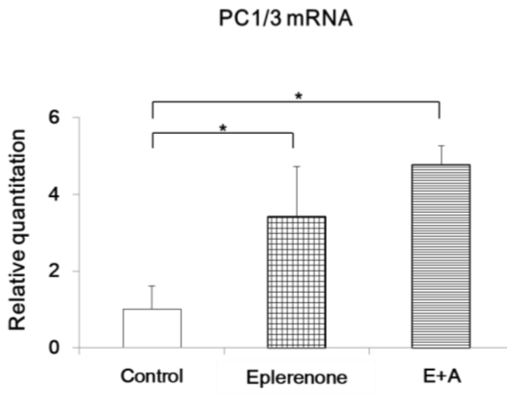

D

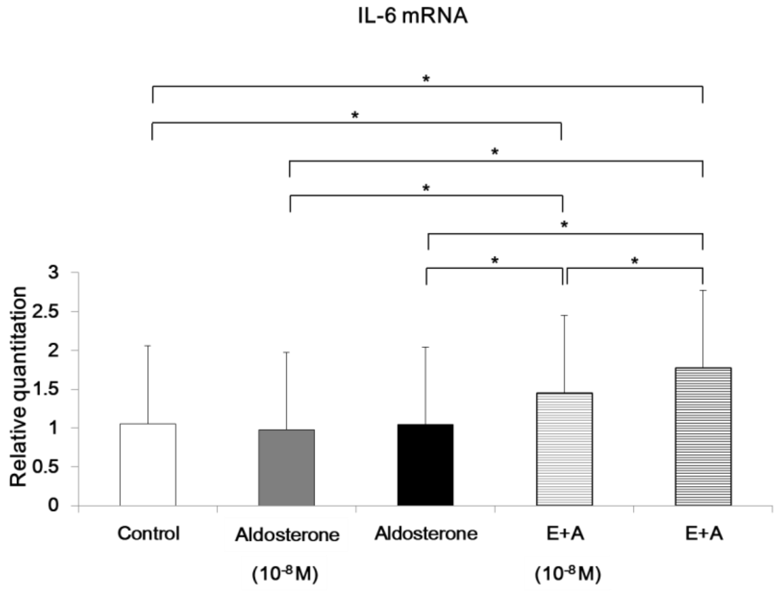

Figure S3

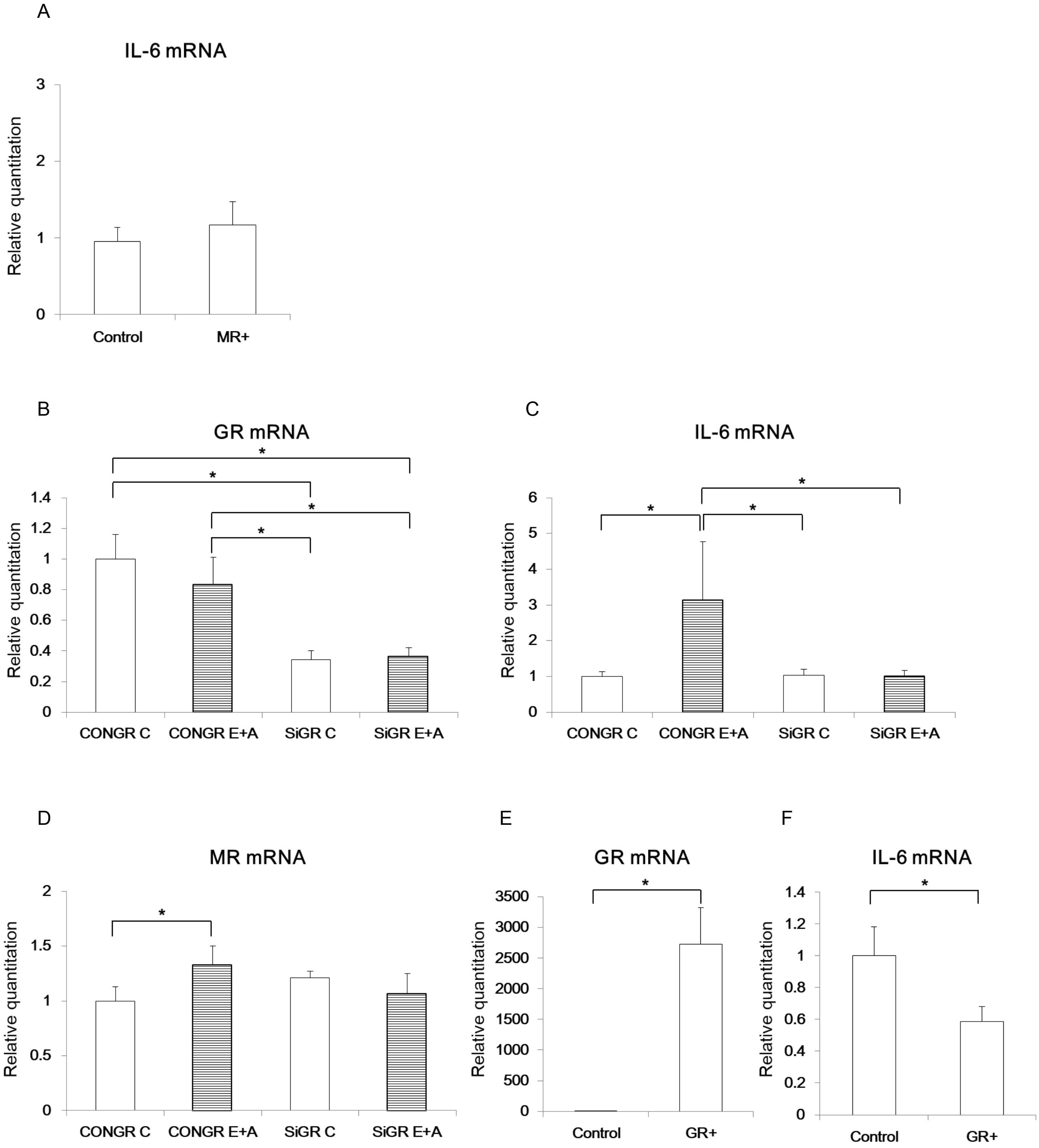
A
IL-6 mRNA
Relative quantitation
3
2
1
0
Control
MR+
B
GR mRNA
Relative quantitation
1.4
1.2
1
0.8
0.6
0.4
0.2
0
CONGR C
CONGR E+A
SiGR C
SiGR E+A
C
IL-6 mRNA
Relative quantitation
6
5
4
3
2
1
0
CONGR C
CONGR E+A
SiGR C
SiGR E+A
D
MR mRNA
Relative quantitation
2
1.5
1
0.5
0
CONGR C
CONGR E+A
SiGR C
SiGR E+A
E
GR mRNA
Relative quantitation
3500
3000
2500
2000
1500
1000
500
0
Control
GR+
F
IL-6 mRNA
Relative quantitation
1.4
1.2
1
0.8
0.6
0.4
0.2
0
Control
GR+

Figure S4

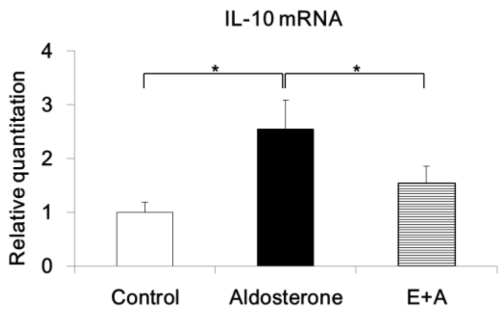
IL-10 mRNA
Relative quantitation
4
3
2
1
0
*
*
Control
Aldosterone
E+A

Figure S5

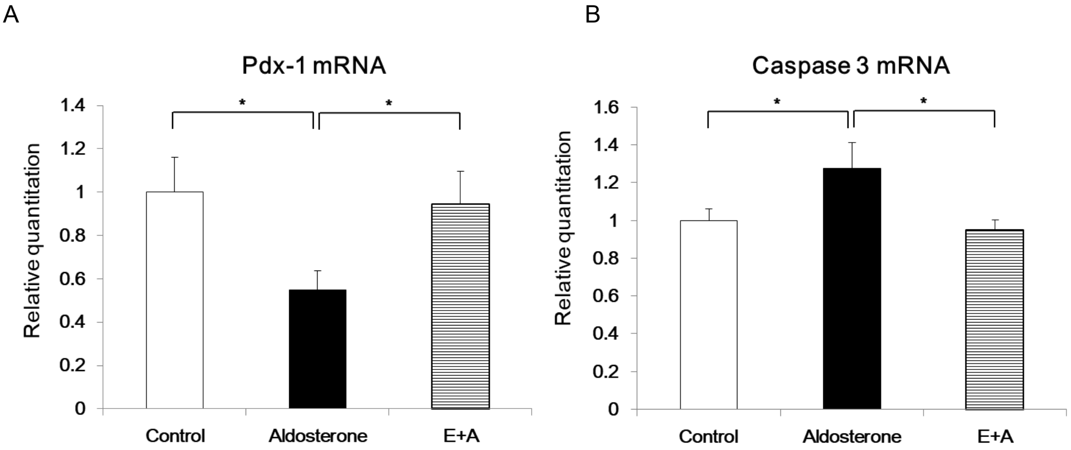
A
Pdx-1 mRNA
Relative quantitation
1.4
1.2
1
0.8
0.6
0.4
0.2
0
*
*
Control
Aldosterone
E+A
B
Caspase 3 mRNA
Relative quantitation
1.6
1.4
1.2
1
0.8
0.6
0.4
0.2
0
*
*
Control
Aldosterone
E+A

Figure S6

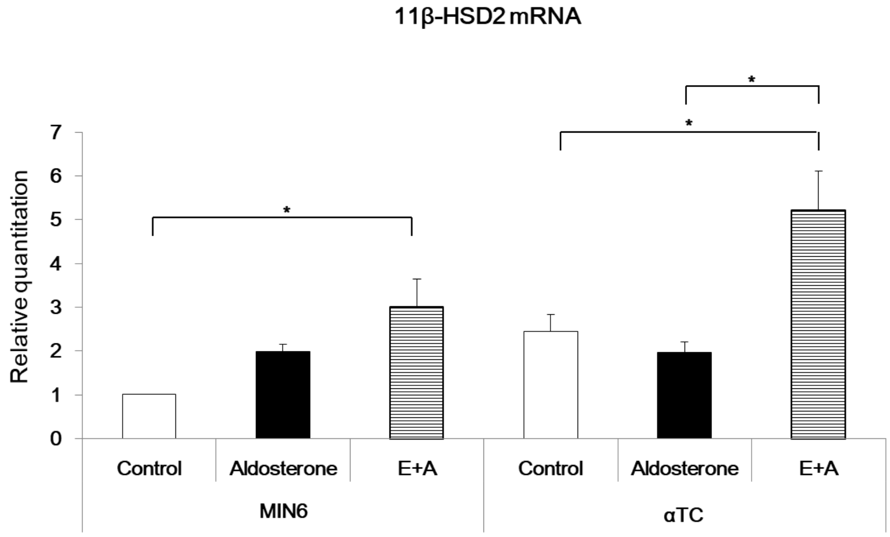

11β-HSD2 mRNA
Relative quantitation
7
6
5
4
3
2
1
0
*
*
*
Control
Aldosterone
E+A
Control
Aldosterone
E+A
MIN6
αTC
